# Unsafe Injection Is Associated with Higher HIV Testing after Bayesian Adjustment for Unmeasured Confounding

**DOI:** 10.34172/aim.2020.113

**Published:** 2020-12-01

**Authors:** Soodabeh Navadeh, Ali Mirzazadeh, Willi McFarland, Phillip Coffin, Mohammad Chehrazi, Kazem Mohammad, Maryam Nazemipour, Mohammad Ali Mansournia, Lawrence C McCandless, Kimberly Page

**Affiliations:** 1 Global Health Sciences, University of California, San Francisco, CA, USA; 2 HIV/STI Surveillance Research Center, and WHO Collaborating Center for HIV Surveillance, Institute for Futures Studies in Health, Kerman University of Medical Sciences, Kerman, Iran; 3 Department of Epidemiology and Biostatistics, University of California, San Francisco, CA, USA; 4 San Francisco Department of Public Health, San Francisco, CA, USA; 5 Division of HIV, ID, and Global Health, School of Medicine, University of California, San Francisco, CA, USA; 6 Department of Epidemiology and Biostatistics, School of Public Health, Tehran University of Medical Sciences, Tehran, Iran; 7 Psychosocial Health Research Institute, Iran University of Medical Sciences, Tehran, Iran; 8 Faculty of Health Sciences, Simon Fraser University, Burnaby, BC, Canada; 9 Department of Internal Medicine, Division of Epidemiology, Biostatistics and Preventive Medicine, University of New Mexico Health Sciences Center

**Keywords:** Bayesian Analysis, Drug injections, HIV, Unmeasured confounder, Unsafe injection

## Abstract

**Background:**

To apply a novel method to adjust for HIV knowledge as an unmeasured confounder for the effect of unsafe injection on future HIV testing.

**Methods:**

The data were collected from 601 HIV-negative persons who inject drugs (PWID) from a cohort in San Francisco. The panel-data generalized estimating equations (GEE) technique was used to estimate the adjusted risk ratio (RR) for the effect of unsafe injection on not being tested (NBT) for HIV. Expert opinion quantified the bias parameters to adjust for insufficient knowledge about HIV transmission as an unmeasured confounder using Bayesian bias analysis.

**Results:**

Expert opinion estimated that 2.5%–40.0% of PWID with unsafe injection had insufficient HIV knowledge; whereas 1.0%–20.0% who practiced safe injection had insufficient knowledge. Experts also estimated the RR for the association between insufficient knowledge and NBT for HIV as 1.1–5.0. The RR estimate for the association between unsafe injection and NBT for HIV, adjusted for measured confounders, was 0.96 (95% confidence interval: 0.89,1.03). However, the RR estimate decreased to 0.82 (95% credible interval: 0.64, 0.99) after adjusting for insufficient knowledge as an unmeasured confounder.

**Conclusion:**

Our Bayesian approach that uses expert opinion to adjust for unmeasured confounders revealed that PWID who practice unsafe injection are more likely to be tested for HIV – an association that was not seen by conventional analysis.

## Introduction

Unsafe drug injection is a major risk factor for HIV and other blood-borne illnesses globally.^1^ In 2014, persons who inject drugs (PWID) accounted for 9% of new HIV diagnoses in the United States, corresponding to nearly 4000 individuals.^2^ Drug injection may promote HIV transmission through associated high-risk sexual behaviors^3^ and through unsafe injection, defined as re-using or sharing needles/syringes.^4^

The first step in the HIV continuum of care, which measures the proportion of persons living with HIV who are diagnosed, requires high coverage and frequent HIV testing in populations at risk.^5^ The “test and treat” prevention strategy aims to reduce HIV transmission through retention and engagement in HIV care of those diagnosed with sustained viral suppression through antiretroviral treatment.^6^ The World Health Organization recommends at least annual HIV testing for PWID.^7^

It has been previously shown that high-risk behavior among key populations may be associated with higher rates of HIV testing. However, these findings have been inconsistent in different high-risk populations, and the reason for this relationship is poorly understood.^8^ The decision to seek HIV testing among PWID depends on knowledge of the risk for HIV through unsafe injection^8^ and testing coverage varies by age,^9^ gender, education, and marital status.^10^

**Figure 1.**
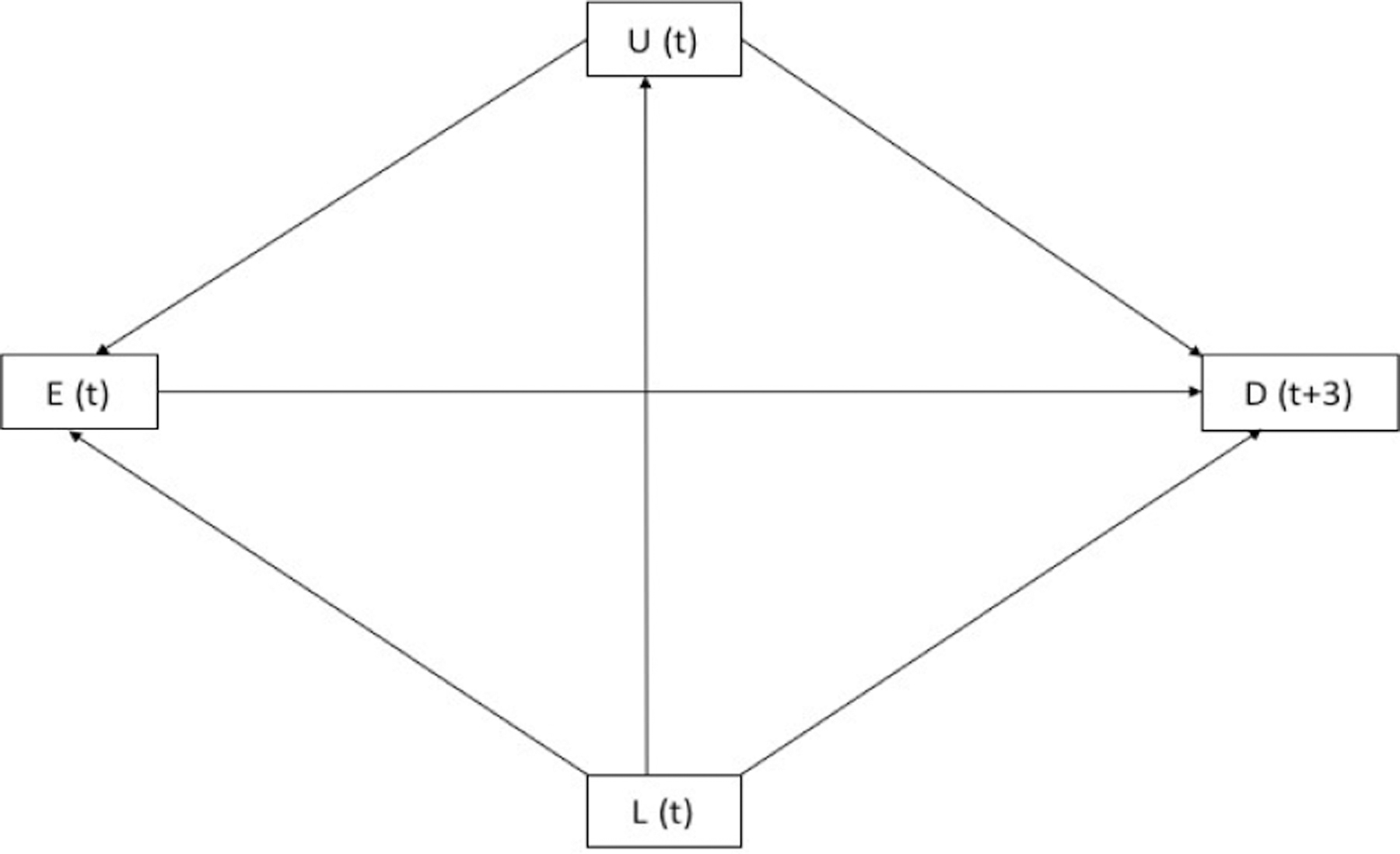
Causal Diagram for the Hypothesized Relationship Between E (Unsafe Injection), D (NBT for HIV), L (Measured Confounders Including Age, Gender, Education, History of Imprisonment at Baseline and 3-Month Follow-Up and Ever Tested for HIV at Baseline), and U (Unmeasured Confounder Insufficient Knowledge). t, denotes month.

We hypothesize that part of the controversy surrounding the relation between unsafe injection among PWID and not being tested (NBT) for HIV may be explained by confounding variables as represented by the causal diagram^11–13^ in Figure 1. Several studies have identified confounders for the effect of unsafe injection on NBT for HIV, including male gender, lower education, and lower knowledge about HIV transmission by increasing the chance of unsafe injection and decreasing the chance of voluntary HIV testing.^14,15^ History of incarceration may also confound the causal pathway by increasing the risk of engaging in high-risk behaviors, and additionally, improving access to the health services provided in prisons.^16^

Confounding bias^17^ is an important threat to observational studies which can be reduced using restriction or matching in the design stage,^11,18^ or alternatively, by using stratification or adjustment at the analysis stage, provided that the confounding variables have been carefully measured and controlled. Any factors not conceived at the design stage or measured during data collection (hereafter called “unmeasured confounders”) make adjustment impossible in conventional analysis. This limitation to conventional frequentist analytic methods is a common critique of observational studies.^19^ Indeed, virtually any observational study may be rightly or wrongly criticized for failure to adjust for unmeasured confounding.

Several analytical methods are available to adjust for unmeasured confounding variables, including Bayesian methods.^20^ However, many of them have been rarely used in the epidemiological literature. This infrequent use of methods to deal with unmeasured confounding, including Bayesian bias analysis,^21^ is due to at least two factors. First, user-friendly statistical packages for such analyses are not yet available. Second, methods to measure priors and conduct Bayesian analysis have not been simplified for use by most applied public health researchers.

Unsafe injection is the major risk factor for HIV transmission among PWID. Interventions to mitigate unsafe injection and associated harms mainly focus on reducing the frequency of unsafe injection by needle exchange programs.^3^ Those who continuously have unsafe injection should be tested more frequently so that they are diagnosed in a timely manner to prevent further HIV transmission. Timely HIV testing, diagnosis and treatment, such as test and treat strategy,^5^ have been shown to reduce HIV transmission in PWID communities. Increasing knowledge about HIV transmission, testing and preventions may play such a role in increasing HIV testing.

In this paper, using empirical data from an ongoing prospective study of young adult PWID in San Francisco,^22,23^ we provide a simple case study to illustrate how to collect and summarize prior bias information. The causal question of interest is to estimate the 3-month likelihood of not receiving an HIV test, if all PWID were low-risk drug injectors, compared with the 3-month likelihood of not receiving an HIV test if all PWID were unsafe drug injectors. We used Bayesian methods to adjust for a hypothetical unmeasured confounder, in this case insufficient knowledge about HIV transmission. The analysis methodology developed for this case study can be used in other studies with few modifications.

## Materials and Methods

### Data Source: The U-Find-Out (UFO) Study

To assess the association between unsafe injection and NBT for HIV, we used data from the UFO Study, an ongoing longitudinal cohort of hepatitis C virus (HCV) uninfected young adult PWID who were under age 30 at recruitment in San Francisco, California, and established in 2000.^22,23^ The study procedures, briefly described below, can be found in detail in prior publications.^23^

### Participants

Young adult PWID recruited by community-based outreach were interviewed for demographic characteristics, drug injection history and sexual risk at enrollment at follow-up visits (for the 3-month period preceding the visit), and tested for HCV infection at enrollment and among negatives at follow-up visits which were scheduled quarterly. HIV testing is offered at each visit, but it not required. Pre- and post-risk-based counseling accompanies both HCV and HIV testing. We analyzed data from 601 HCV/HIV-negative PWID with at least one follow-up visit.

### Variable Definitions

At each visit, a trained interviewer asked about recent HIV testing and injection behavior in the previous three months. We categorized PWID into unsafe injectors and low-risk injectors. PWID who shared, reused, or borrowed previously used drug-preparation equipment in the three months before the interview were defined as unsafe injectors.

Measured confounders included sex (male, female, transgender), age, education, having been incarcerated and ever having tested for HIV at the time of baseline interview. In our external bias adjustment, we considered insufficient knowledge about HIV transmission routes as the potential unmeasured confounder. The insufficient HIV knowledge is clearly a confounder in our setting as it positively affects both unsafe injection and NBT. In other words, insufficient HIV knowledge is a common cause of the exposure and outcome and so, it is a causal or classical confounder.^24,25^ The causal diagram in Figure 1 represents the causal relationship between variables within the population in two successive visits.

We defined insufficient knowledge about HIV transmission as not knowing the following forms of prevention: consistent use of condom, having only one uninfected faithful partner and not sharing syringe/needle, and additionally, not rejecting two misconceptions: (i) knowing that healthy looking persons can be HIV positive; and (ii) knowing that HIV cannot be transmitted from sharing food or mosquito bites.^26,27^

### Conventional Analysis

Since each PWID may have had more than one visit during the study period, we applied a generalized estimating equations (GEE) methodology to assess the association between unsafe injection and NBT for HIV, after adjusting for measured confounders. PWID were censored at the first visit in which they reported having been HIV tested and seroconverted or self-reported HIV-positive, and otherwise were followed up to the last visit that HIV testing data were available. We considered unsafe injection with one interval lag (three months) as the main exposure prior to the measurement of the outcome variable (reporting having had an HIV test). Therefore, for every visit, the effect of unsafe injection in the previous visit was assessed on NBT for HIV history measured at that visit. In other words, we assigned one visit lag interval between the exposure at visit t and the outcome at visit t+1. Since each participant had more than one measurement of the outcome, we used GEE with Poisson distribution, logarithmic link function, and cluster robust standard errors to estimate the Risk Ratio (RR) and 95% confidence interval (CI). For this analysis, we used the Xtgee command in Stata software version 14.0 (StataCorp, College Station, TX, USA). For comparison, we also repeated the analysis using a random-effect Poisson regression analysis using Xtpoisson command in Stata.

### Bias Analysis: Prior Values and Distributions

To account for insufficient knowledge about HIV transmission routes as the unmeasured confounder, we used a Bayesian bias analysis.^19,20,28–31^ We used expert opinion to derive prior probability distributions for three bias parameters that characterize the magnitude and direction of unmeasured confounding.^21^ That is, we approached two experts with expertise in the epidemiology of HIV and drug use/injection in San Francisco to give us 95% prior intervals for the following bias parameters; see the questionnaire in the Supplementary file 1:

- Proportion of young adult PWID with insufficient HIV knowledge among unsafe injectors (P_1_)
- Proportion of young adult PWID with insufficient of HIV knowledge among low-risk injectors (P_0_)
- RR for the association between insufficient HIV knowledge and NBT for HIV among PWID who practice unsafe injection (RR_UY_)

We then assigned prior probability distributions to each of the bias parameters based on the prior intervals suggested by the experts; beta distributions were used for the two proportions in P_1_ and P_0_, and normal distribution for the logarithm of RR_UY_.^21^

The beta distribution was used for the bias parameters P_1_ and P_0_ as it is a conjugate prior for the Bernoulli distribution: considering a Bernoulli distribution for the data, which is natural for binary variables like insufficient knowledge, and a beta distribution as the prior for the proportions (like P_1_ and P_0_), the posterior will also be a beta distribution. For beta distribution used for P_1_ and P_0_, we selected alpha and beta values so that the percentile 2.5 and 97.5 of beta distribution exactly match the 95% prior intervals of the experts using a grid search. For normal distribution considered for ln(RR_UY_), the mean was calculated by averaging the logarithm of upper and lower prior limits, and standard deviation was computed by subtracting logarithm of lower limit from logarithm of upper limit and then dividing by 3.92.

### Bayesian Bias Analysis for Unmeasured Confounding

Markov chain Monte Carlo (MCMC) using Gibbs sampling was used to sample from the posterior distribution. In each MCMC iteration, the posterior RR for exposure, adjusted for measured confounders, was estimated. A Gaussian random-effect Poisson model was used to assess the effect of unsafe injection on NBT for HIV adjusted for measured confounders. We assigned an uninformative normal distribution, with mean and variance equal to zero and 10^6^ respectively, for the regression coefficients for the exposure variable and other covariates in the model for NBT for HIV. The Gaussian random effects were assigned a normal distribution with mean zero and variance equal to τ, where 1/τ was assigned a gamma (0.001, 0.001) distribution. The inverse-gamma distribution is frequently used for variance parameter as it is conditionally conjugate in the sense that if variance has an inverse-gamma prior distribution, then the conditional posterior distribution is also inverse-gamma.^32^ In the next step, a sample was drawn using beta and normal distributions of bias parameters and to estimate the bias correction factor, and adjusted RR for insufficient knowledge about HIV transmission routes was derived from unadjusted RR (adjusted only for measured confounders) divided by bias correction factor^20^:

$$\text{Bias correction factor}=\frac{{\text{RR}}_{\text{UY}}{\text{P}}_{1}+\left(1-{\text{P}}_{1}\right)}{{\text{RR}}_{\text{UY}}{\text{P}}_{0}+\left(1-{\text{P}}_{0}\right)}$$

$$\text{Adjusted RR=}\frac{\text{Unadjusted RR}}{\text{Bias correction factor}}$$

The models converged after 4000 iterations with 1000 iterations of burn-in, and convergence was assessed using the Monte Carlo standard error. After convergence was achieved, we ran the simulation until the Monte Carlo error for adjusted RR became less than 5% of the sample standard deviation. This occurred at 10 000 iterations.^33^ We used WinBUGS (version 1.4) for the bias analysis, and the WinBUGS computer code is given in Supplementary file 2. We reported the RR with 95% posterior credible interval (median and 2.5^th^ and 97.5^th^ percentiles of posterior distribution) using the two experts’ 95% prior intervals separately.

## Results

Our analytical sample included 601 persons with an average of 4.3 visits (SD = 7.7). At the baseline visit, nearly two-thirds (65.9%) reported unsafe injecting in the previous three months and only two individuals did not answer this question. The frequency of unsafe injection was significantly higher in women than in men (75.0% vs. 61.4%, *P* = 0.003). Unsafe injection did not significantly vary by the other baseline covariates (history of incarceration, lower education, and ever tested for HIV). The mean (SD) age was approximately 23.6 (3.4) years, regardless of injection behavior (Table 1).

Table 2 shows the distribution of the three bias parameters based on the opinion of two experts. The experts estimated that the proportion of PWID with insufficient knowledge was between 2.5% and 40.0% among unsafe injectors, and between 1.0% and 20.0% among low-risk injectors. Accordingly, for expert 1, we assigned a beta (8.65, 149.42) prior to parameter ‘a’, and a beta (5.12, 166.35) prior to parameter ‘b’, which yield suitable 95% prior probability credible intervals for the proportions based on the first expert opinion. In contrast, expert 2 held more extreme views about the magnitude of unmeasured confounding. The characteristics of the beta distribution that were assigned are shown in Table 2. Additionally, the lower bound for the RR of the association between insufficient knowledge and NBT for HIV was estimated as 1.1 by both experts and the upper bound that was either 2.2 or 5.0. The corresponding normal distributions are presented in Table 2.

The results of the analyses are given in Table 3. The RR for the association between unsafe injecting and NBT for HIV, adjusted for measured confounders (age, gender, education, history of imprisonment at baseline and in 3-month follow up and reporting ever testing for HIV at baseline) was 0.96 (95%CI, 0.89–1.03). After adjusting for the unmeasured confounder, insufficient HIV knowledge, the RR between unsafe injection and NBT for HIV (adjusted for both measured and unmeasured confounders) decreased to 0.95 (95% credible interval, 0.84–1.08) in the first bias analysis and 0.82 (95% credible interval, 0.64–0.99) in the second bias analyses. In other words, the bias analysis revealed that PWID who are practicing unsafe injection are more likely to be tested for HIV.

## Discussion

We found that if the insufficient HIV knowledge was considered as an unmeasured confounder, then PWID who practice unsafe injection are more likely to be tested for HIV – an association that was not seen by conventional analysis. The approach can be used as a post-hoc correction when experts, peer-reviewers, or other emerging data deem that biases are possible or likely, as in the present case.^10^ The negative effect of unsafe injection on NBT can be explained by the notion that people with unsafe injection are more reactive to receiving knowledge about the transmission routes of HIV which in turn encourages them to refer more for HIV testing. In this sense, the unmeasured variable HIV knowledge mediates the effect of unsafe injection on NBT. In fact, depending on its measurement time (before or after the unsafe injection), HIV knowledge can act as a mediator or confounder. Our results indicated that adjustment for unmeasured insufficient HIV knowledge intensifies the relation between exposure and outcome. In fact, the relation between exposure and outcome through the confounder is positive, a positive multiplied by a positive is positive, but the effect estimate of the exposure is negative (adjusted RR = 0.95 and 0.82 based on inputs of expert 1 and 2). So, we expect the unadjusted RR, which involves some cancellation of these two relationships, will be closer to the null than the adjusted one.^34^

To better understand how Bayesian bias analysis for unmeasured confounding works, we can plug the typical values, arithmetic mean of prior limits for P_1_ and P_0_ and geometric mean of prior limits for RR_UY_, provided by expert 1 in the bias correction factor and then obtain the adjusted RR. Based on Table 2, ${\text{P}}_{1}=\frac{0.2+0.4}{2}=0.3$, ${\text{P}}_{0}=\frac{0.1+0.2}{2}=0.15$ and $\text{RR}=\sqrt{1.1*5}=2.35$; so, $\text{bias correction factor}=\frac{\left(2.35*0.3\right)+0.7}{\left(2.35*0.15\right)+0.85}=1.17$ and adjusted $\text{RR}=\frac{0.96}{1.17}=0.82$ which is the same point estimate we obtained from Bayesian bias analysis (Table 3).

Since the outcome of interest “NBT for HIV” was not a rare event, we calculated the RR. Estimating odds ratio when the outcome is not a rare event may result in misinterpretation, i.e. odds ratio exaggerates the results compared with RR and it suffers from non-collapsibility.^35–38^ The GEE method estimates the marginal effect, whereas the random-effect Poisson model used in the Bayesian analysis estimates the conditional effects (conditional on the cluster-specific random term). We performed the conventional random-effect Poisson regression analysis using both Stata and WinBUGS (without considering the unmeasured confounder, and with uninformative priors for beta coefficients) and obtained the same results of GEE which is not surprising given the collapsibility of RR.^35^

Although Bayesian analysis can be done by just one set of priors that derives from expert opinion,^20^ overconfidence of experts is an important issue and can affect the analysis results and conclusion.^39^ Because the parameters for unmeasured confounding are not identifiable using data, the results strongly depend on the choice of prior distribution. For example, the prior distributions for the prevalence of unmeasured confounder among exposed and unexposed suggested by two experts were very different and did not even overlap with each other. It means that at least one of the priors is wrong. To overcome this problem, it is better not to rely on only one or two experts. Instead, researchers should ideally collect data from several experts (and/or through a comprehensive review of the literature if it exists) and synthesize the results to reach a unique set of priors.^21^

The results from the two bias analyses based on the first and second expert opinions were different. The difference was due to the differences in the prior distributions provided by the two experts. In particular, the priors of the expert 1 seem to suffer from overconfident (overly precise) bias,^40^ strongly suggesting that the bias parameters (P_1_, P_0_, RR_UY_) are small and so insufficient HIV knowledge is a very weak confounder.

Our study has some limitations other than using only two experts. First, we use self-reported data on exposure, outcome and measured confounders. Although most behavioral surveys rely on self-reported measures of risk behaviors, they are prone to recall and social desirability biases.^41–43^ Second, we only looked at one unmeasured confounder and there might be other individual and population-level confounders for NBT for HIV that we did not assess. For example, no information was available about a history of mental illness or unstable housing (e.g. homelessness), and both these factors have been shown to impact access to HIV care.^44^ However, adjusting for several correlated unmeasured confounders requires strong untestable modelling assumptions, and is an ongoing area of statistical research.^45^

The best way to deal with unmeasured confounding is to measure and adjust for all important confounders. In the present study, however, unsafe injection is a time-varying exposure and insufficient knowledge about HIV transmission routes is indeed a time-varying confounder (as presented in Figure 1). Moreover, there may be feedback between unsafe injection and insufficient knowledge (i.e., they may affect one another). Consequently, insufficient knowledge about HIV transmission routes may be a time-varying confounder affected by the previous exposure, and causal methods are required to support a valid analysis.^39,46–55^

In addition to the bias analysis method we used to address unmeasured confounding, our study emphasizes the importance of sufficient HIV knowledge about HIV transmission routes on NBT for HIV in PWID. Interventions to increase the knowledge of PWID and other high-risk populations may increase the HIV testing rate and the chance of early HIV diagnosis.

In conclusion, Bayesian bias analysis for unmeasured confounding adjustment can be accomplished using a set of priors derived from the expert opinion and translating them to the bias parameters for estimating the bias correction factor. The adjusted RR for unmeasured confounder equals unadjusted RR divided by bias correction factor. Our Bayesian approach that uses expert opinion to adjust for unmeasured confounders revealed that PWID who practice unsafe injection are more likely to be tested for HIV – an association that was not seen by conventional analysis.

## Supplementary Material

Supp 1 Supplementary file 1. Questionnaire for Eliciting the Study Priors.

Supp 2 Supplementary file 2. WinBUGS code.

## Figures and Tables

**Table 1.** Baseline Characteristics of People Who Inject Drugs by their Injection Risk Behavior, UFO study, San Francisco

Characteristics	No Recent Unsafe Injection; n = 204 (%)	With Recent Unsafe Injection; n = 395 (%)	*P* Value*
Gender			
Male	151 (38.62)	240 (61.38)	
Female	51 (25)	153 (75)	0.003
Transgender	2 (50)	2 (50)	
Any history of incarceration			
No	148 (34.66)	279 (65.34)	0.47
Yes	52 (31.52)	113 (68.48)	
Education			
Less than high school	74 (32.17)	156 (67.83)	0.43
High school and up	129 (35.34)	236 (64.66)	
Ever tested for HIV			
Yes	60 (35.29)	110 (64.71)	0.67
No	141 (33.49)	280 (66.51)	
Age; mean (SD)	23.8 (3.2)	23.5 (3.5)	0.21

SD, standard deviation.

* *P* values were driven from chi square test except for age that was driven from independent *t* test.

**Table 2.** Expert Opinions About the Three Bias Parameters and the Assigned Distributions

Bias Parameters	95% Prior Interval (by Expert Opinion)		Prior Distributions	

	Expert 1	Expert 2	Expert 1	Expert 2
a) Proportion of young adult PWID with insufficient HIV knowledge among unsafe injectors	2.5%–9.5%	20%–40%	Beta distribution (α = 8.65, β = 149.42)	Beta distribution (α = 23.11, β = 55.21)
b) Proportion of young adult PWID with insufficient HIV knowledge among low-risk injectors	1%–6%	10%–20%	Beta distribution (α = 5.12, β = 166.35)	Beta distribution (α = 27.83, β = 162.15)
c) Risk Ratio of the association between insufficient HIV knowledge and NBT for HIV among low-risk injectors	1.1–2.2	1.1–5	Normal distribution (μ = 0.44, σ = 0.18)	Normal distribution (μ = 0.85, σ = 0.39)

**Table 3.** Risk Ratio Between Unsafe Injection and NBT for HIV after Adjusting for Measured and Unmeasured Confounders

	Risk Ratio	95% Confidence/Credible Interval
Unsafe injection	0.96^a^	0.89–1.03^b^
Adjusted unsafe injection Bias analysis 1 (expert 1)	0.95^c^	0.84–1.08^d^
Adjusted unsafe injection Bias analysis 2 (expert 2)	0.82^e^	0.64–0.99^d^

a Risk Ratio adjusted for the measured confounders (age, gender, education, history of imprisonment at baseline and 3-month follow-up and ever tested for HIV at baseline).

b 95% Confidence Interval.

c Risk Ratio adjusted for measured confounders (age, gender, education, history of imprisonment at baseline and 3-month follow-up and ever tested for HIV at baseline) and unmeasured confounder (insufficient knowledge) based on the first expert’s priors.

d 95% Credible Interval.

e Risk Ratio adjusted for measured confounders (age, gender, education, history of imprisonment at baseline and 3-month follow-up and ever tested for HIV at baseline) and unmeasured confounder (insufficient knowledge) based on the second expert’s priors.
