# Supplementary material for: Unsafe Injection Is Associated with Higher HIV Testing after Bayesian Adjustment for Unmeasured Confounding
Source: Arch Iran Med. Author manuscript; Available in PMC 2023 Jan 17. (PMC9844981; doi:10.34172/aim.2020.113)
Supplement: Supp 1 — Supplementary file 1. Questionnaire for Eliciting the Study Priors. [file NIHMS1858975-supplement-Supp_1.pdf]

Supplementary file 1. Questionnaire for Eliciting the Study Priors.

Q1. Based on your opinion, what proportion of PWIDs with high-risk injection had insufficient knowledge about HIV transmission routes? Your answer should be between 0-100%.

Point Estimate ……

Lower Bound ……..

Upper Bound ……..

Q2. Based on your opinion, what proportion of PWIDs who **did not** have high-risk injection had insufficient knowledge about HIV transmission routes? Your answer should be between 0-100%.

Point Estimate ……

Lower Bound ……..

Upper Bound ……..

Q3. We hypothesize that lack of knowledge about HIV transmission routes is a predictor for HIV testing in PWIDs and possibly confounds the association between having history of high-risk injection and HIV testing. Based on your opinion, how many times higher or lower is the chance of **not doing** the HIV test among people with lack of knowledge than people who have enough knowledge about HIV transmission routes? In other words, what is the risk ratio (RR) of the association between knowledge about HIV transmission routes and HIV testing (the answer should be >0)?

Point Estimate ……

Lower Bound ……..

Upper Bound ……..
