# Supplementary material for: Unsafe Injection Is Associated with Higher HIV Testing after Bayesian Adjustment for Unmeasured Confounding
Source: Arch Iran Med. Author manuscript; Available in PMC 2023 Jan 17. (PMC9844981; doi:10.34172/aim.2020.113)
Supplement: Supp 2 — Supplementary file 2. WinBUGS code. [file NIHMS1858975-supplement-Supp_2.pdf]

Supplementary file 2. WinBUGS code.

Bayesian Poisson model with adjustment for unmeasured confounder (insufficient knowledge about HIV transmission routes) based on the second expert's priors.

```
### α: NBT for HIV; β: high risk injection; γ: history of imprisonment in the past 3 months; δ: age; ε: education; η: gender; λ: history of imprisonment in the past 3 months at baseline; φ: HIV testing at baseline ###

model {

for(i in 1:N) {

α[i]~dpois(mu[i])

log(mu[i])<-b0+b1* β[i]+b2* γ[i]+b3* δ[i]+b4* ε[i]+b5* η[i]+b6* λ[i]+b7* φ[i]+u[group[i]]

}

##M Groups

for (j in 1:M) {

u[j] ~ dnorm(0.0,τ) }

b0~ dnorm(0.0,1.0E-6)

…

b7~ dnorm(0.0,1.0E-6)

1/τ~ dgamma(0.001,0.001)

### Estimation of the relation between insufficient knowledge and NBT for HIV (RR)

##ω~ dnorm(mu, percision)

ω~ dnorm(0.85237405, 6.7026589)

### Estimation of insufficient knowledge prevalence in the exposed and unexposed strata

π~ dbeta(24.20208, 56.47152)

ρ~ dbeta(29.38824, 166.53336)

### Estimation of unknown bias correction factor

bias<-((1- π)+(exp(ω)*( π)))/((1- ρ)+(exp(ω)*( ρ)))
```

```
###Estimation of risk ratio uncorrected for confounding by insufficientknowledge about HIV transmission routes

RR<-exp(b1)

### Estimation of risk ratio adjusted for insufficient knowledge about HIV transmission routes

RR.adj<-RR/bias

}

# Specification of Initials

list(b0=0, b1=0, b2=0, b3=0, b4=0, b5=0, b6=0, b7=0, ω =0, π =0, ρ =0, τ=1,

u=c(0, 0, …., 0))
```
